# The effect of Flipped Classroom through Near Peer Education (FC through NPE) on patient safety knowledge retention in nursing and midwifery students: a solomon four-group design

**DOI:** 10.1186/s12909-022-03144-w

**Published:** 2022-02-19

**Authors:** Sima Poormahdi Golaki, Farahnaz Kamali, Razieh Bagherzadeh, Fatemeh Hajinejad, Hakimeh Vahedparast

**Affiliations:** 1 Student Research Committee, Nursing and Midwifery Faculty, Bushehr University of Medical Sciences, Bushehr, Iran; 2 Department of Midwifery, Nursing and Midwifery Faculty, Bushehr University of Medical Sciences, Bushehr, Iran; 3 Department of Nursing, Nursing and Midwifery Faculty, Bushehr University of Medical Sciences, Salmanefarsi Blvd, Bushehr, Iran

**Keywords:** Flipped Classroom, Near-Peer Education, Patient Safety, Nursing, Midwifery, Students, Knowledge Retention

## Abstract

**Introduction:**

Selecting an appropriate teaching methodology is one of the key stages in education. This study is an attempt to delve into the effect of FC through NPE on patient safety knowledge retention in nursing and midwifery students.

**Methods:**

A randomized controlled trial, using the Solomon design, was conducted in 2019 on 82 nursing and midwifery students enrolled from Bushehr nursing and midwifery school. The Subjects were then allocated to four groups via block randomization. The Subjects in both intervention groups studied the educational content online for 2 weeks and subsequently attended the FC through NPE. Both control groups merely received education based on conventional method. The post-test was once administered to the four study groups immediately after completing the program and once again 2 months after it.

**Results:**

The posttest mean scores of knowledge retention in both intervention groups remained the same (*P* = 0.1), while they were higher in the control groups (*P* < 0.05). The changes in the mean scores of the post-test in the intervention and follow-up groups did not demonstrate a statistically significant difference between the four study groups (*P* = 0.130, F = 1.941).

**Conclusion:**

The use of the FC through NPE increased the knowledge mean scores; however, it failed to affect knowledge retention. Given the infancy of this pedagogical approach, further studies are needed to investigate its effects on various learning outcomes.

## Background

Innovations and rapid changes in socioeconomic, political, and technological domains have mostly challenged medical education with their own complexities. They have additionally made the roles of medical science educators much more prominent and more complicated than ever. Moreover, it no longer seems possible to lead medical students toward progress via conventional methods [1].

In the present era, selecting the most appropriate teaching methodologies is one of the strategies adopted by teachers to augment the quality of their education according to students’ specific goals and conditions [2]. Hence, using new teaching methodologies is among one of these strategies. The FC approach is thus assumed as one of the new pedagogical models, *which is dependent on technology* [3], even if such methods have created several challenges in education, especially in medical sciences [4–8].

Some studies have further supported the FC as a pedagogical approach for independent learning [4], which results in more learning compared with other methods [8], and can even produce high levels of satisfaction in learners [7]. Nevertheless, there has also been evidence of lack of satisfaction on the part of the learners [8] and the same effects as other teaching methods [9]. The results of a review on nursing education indicated contradictory results in relation to the effect of using the FC on the levels of satisfaction, learning rate, and grade point average in students [5, 8]. Existing evidence also leads to the assumption that a pedagogical model, even a new one alone, cannot advance learning, so an integration of several methods should be exercised to create active learning and thinking in learners [6]. It seems that the integration of two new pedagogical methods can cover the weaknesses of each other. In this sense, the NPE, as one of the effectively positive pedagogical methods, is often evaluated by medical students [10], because it is believed that peers are more familiar with their own educational needs and can better understand those of other students and help them learn [11].

Patient safety (PS), as one of the priorities of global health, is an important issue in medical education that requires the use of long-lasting methods of teaching. Accordingly, utilization of pedagogical strategies is a key element in improving attitudes and practices associated with PS. The NPE has been thus recognized formally and informally by many organizations as an effective way to provide PS education [3, 12]. Although most studies have not so far assessed knowledge retention [13–16], their results show that this type of education can prove effective in PS [14], by improving attitudes toward PS in students [16], thus enhancing their chances of success in medical education programs [17].

Unfortunately, many studies on medical students have demonstrated that they receive insufficient education to increase support capabilities to ensure PS and have even gone so far to claim that they lack the necessary knowledge or skills [1]. Since the world of education today has shifted its focus from teaching to learning [17], medical science education including nursing and midwifery requires fundamental changes [18]. A review of the literature shows that new methods of teaching are not entirely desirable and common, either [19]. For this reason, education specialists seek to exploit different teaching methodologies simultaneously [20] to remove their limitations and disadvantages. Therefore, considering the importance of knowledge retention in PS education for nursing and midwifery students and the irreparable harm caused by students’ insufficient knowledge of PS, an integration of two new methods of teaching to cover the weaknesses of each other seems of utmost importance. The present study was undertaken as an attempt to compare the effect of FC through NPE and conventional methods on PS knowledge retention in nursing and midwifery students enrolled in Bushehr University of Medical Sciences, Bushehr, Iran.

## Methods

### Study design

This study was a randomized controlled trial (IRCT code: IRCT20090522001930N3, 18/3/2019**)** with a pretest-posttest and follow-up as well as intervention and control groups. The Solomon four-group design was utilized to remove the effects of the pretest in sensitizing the students and preventing damage to the external validity of the study. In this sense, the samples were randomly divided into four groups, i.e., two intervention groups and two control groups. Then, only the pretest was administered to one intervention group and one control group, and then all four groups received a post-test/follow-up knowledge retention test.

### Subjects

The nursing and midwifery students in their third-semester were enrolled in the study and were divided into four groups. As the total number of the students in the classrooms was 92, 23 subjects were considered for each group, of whom 46 were exposed to the conventional method and 46 were exposed to the FC through NPE setting. The subjects were then divided into four groups via block randomization. First, the groups were determined, with A and B representing the intervention groups 1 and 2, and C and D representing the control groups 1 and 2, respectively. Then, blocking was performed using the random allocation software. To have an equal proportion of the nursing and midwifery students in each group, they were blocked separately. Since there were 64 nursing and 28 midwifery students, eight 8-part blocks and seven 4-part blocks were considered, respectively. Students were assigned to the blocks based on the attendance list that was obtained from the faculty’s education department. Block randomization was carried out by one of the researchers (R.B.), who was unaware of the allocation of the students to intervention and control groups. None of the students knew to which group they belonged before the program began.

The inclusion criteria in this study included students enrolled in the third semester of nursing and midwifery program, having access to a computer or smartphone, and willingness to participate in the research. On the other hand, the exclusion criteria were absenteeism for any reason, not viewing the educational content, and unwillingness to continue with the study.

### Conventional method

**Fig. 1:**
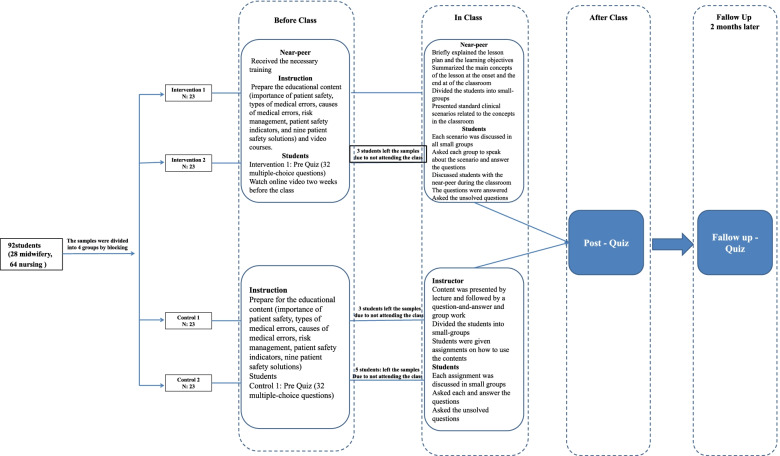
A summary of the study in the form of a flowchart

The control group was taught in the context of a workshop. The workshop group consisted of three two-hour sessions held in 1 day. PPTs were used to explain the teaching contents. The lecturing method was the predominant approach used in the workshop, followed by a question-and-answer period and group work. The teaching contents for the control groups were the same as for the intervention groups. Fig. 1 illustrates a summary of the study in the form of a flowchart.

### FC through NPE method

The teacher prepared the content before the class. The PPTs and the Camtasia Studio software were also employed to prepare the educational content and to present it to the intervention groups. The content was then uploaded to a website specifically designed for this purpose 2 weeks before the implementation of the FC through NPE, and the members of the intervention groups were asked to study the full course content within 2 weeks before the class began. After browsing the website, the users were greeted with a welcome message that asked them to create their personal information page by typing in their usernames and passwords. Due to the interactive design of the website, the students in both intervention groups could access it as users at any time and place by connecting to the Internet with their smartphones, tablets, and personal computers and view the educational content uploaded on the website.

The researchers could quickly check the latest follow-up status of the course completed by the students in the intervention groups from the management panel. In addition, further follow-ups were done, when necessary, to encourage the students to study the educational content by sending messages or making phone calls.

The near peer (as a master’s student in nursing) also received the necessary training in three two-hour sessions before implementing the FC under the supervision of the relevant educator. To avoid any errors in conveying the educational concepts, the near peer could lead the classroom in the presence of the educator. The educational content was then presented in the form of three two-hour sessions during 1 day. The near peer divided the students into groups of five or six, and the students were then asked to sit next to each other with their counterparts in the same group. The near peer briefly explained the lesson plan and the learning objectives at the onset of the class and summarized the main concepts of the lesson at the end. The standard clinical scenarios related to the concepts were drawn from a book published by the Ministry of Health and Medical Education, entitled as *Patient Safety* [21], prior to the intervention, and were then presented in the classroom. Next, the learners were given 5 min to analyze each scenario with all their group members and answer the questions corresponding to each scenario. Afterwards, each group was asked to speak about the scenario and answer the questions, discuss them with the near peer and other students during the classroom, and analyze and answer the questions related to the clinical scenarios, to become familiar with the application of theoretical knowledge in clinical practice.

### Intervention

Upon explaining the research procedure and receiving the informed consent form, the pre-test was administered to the intervention group one and the control group one. The control group received the PS program training via the conventional method, while the interventional group engaged in the FC through NPE approach. Conventional teaching (for the control groups) was done first and the FC through NPE teaching was done later to minimize the direct effect of flipped teaching method on the control group. After the class was over, all the students in the four groups were tested, and 2 months after the program, all the four groups received a posttest to assess their knowledge retention. The subjects were administered the same test, which was designed by the researchers to evaluate the levels of knowledge in all the three stages, i.e., before, immediately after, and 2 months after the intervention. It should be mentioned that only identity numbers were used instead of real names in the tests. It was emphasized that *test scores* had no effect on grades or performance in the class. All the students were unaware of their assignments and scenarios before the program. To evaluate the learning outcomes, the test was carried out by one of the examination committee members, who was on the research team. Fig. 1 is a flowchart that summarizes the FC through NPE and the conventional method.

The content of the PS education program was also prepared based on the standard principles of PS provided by the World Health Organization (WHO) [22], scientific references [23, 24], as well as circulars of Iran’s Ministry of Health and Medical Education [21]. The educational content included information about the basic concepts of patient safety, types of medical errors, risk management, qualities of effective team players, patient safety activities, as well as nine patient safety solutions. All the subjects received the same content, the same syllabus, and the same practical guidance. Table 1 illustrates the content of the PS education program and a summary of the related details.

**Table 1:** The content of the patient safety education program

Topic	Detail Contents
Basic Concepts of Patient Safety	Introduction of patient safety and laws
	Introduction of medical institution certification mark
	Importance of patient safety
	Definitions & incidence of medical errors
Types of Medical Errors	Medication events
	Healthcare-associated infections
	Surgical errors
	Laboratory errors
	Documentation
	Patient Falls
	Pressure ulcer
Risk Management	Identify the risk
	Risk analysis (Quantify & Prioritize Risk)
	Strategies to reduce, eliminate or transfer risk
	Continuous monitoring
	*Effective communication*
Being an Effective Team Player	Team & Values, roles and responsibilities
	Learning styles
	Listening skills
	Team coordination
	Effective leadership
	Characteristics of successful teams
	Effective communication and communication tools
	Conflict resolution
	Evaluation of team performance
Patient safety activities	Learning from errors to prevent harm
	Being an effective team player
	Patient identification
	Bedsores prevention activities
	Fall prevention activities
	Infection prevention activities
Nine Patient Safety Solutions	Look-alike, sound-alike medication names
	Patient identification
	Communication during patient handovers;
	Performance of proper procedures at correct body sites
	Control of concentrated electrolyte solutions
	Assuring medication accuracy at transitions in care
	Avoiding catheter and tubing misconnections
	Single-use of injection devices
	Improved hand hygiene

### Data evaluation

A demographic form and the Patient Safety Knowledge Retention Exam (PSKRE) were used to collect the research data.

The demographic form included items on age, gender, year of admission, being native, place of residence (i.e., dormitory or private home), and field of study.

In order to develop the PSKRE, 70 questions were initially prepared and designed based on blueprints and education objectives associated with PS set by the Ministry of Health and Medical Education. Then, the questions were submitted to 10 faculty members of the School of Nursing and two experts in the field of PS to assess the validity. Afterwards, the questions were reviewed by said faculty members and their opinions were applied in the final draft. Finally, the questions were reduced to include 32 four-option multiple-choice questions that covered all the aspects of knowledge associated with PS.

### Statements

All protocols were carried out in accordance with relevant guidelines and regulations. All *interventional* protocols, specifically, Flipped Classroom by Near Peer Education (and relevant protocols) were approved by the Ethics Committee of Bushehr University of Medical Sciences (Code: 1397.128.IR.BPUMS.REC) and Iranian Registry of Clinical Trials (IRCT20090522001930N3) in 18/3/2019. Informed consent was obtained from all subjects, and all methods were carried out in accordance with the relevant guidelines and regulations.

### Data analysis

The statistical *analyzer (RB)* did not know the identity of any of the subjects, as numbers were used instead of their real names and *the letters of the alphabet were used as identifiers for the* groups*.* To describe the data, descriptive statistics including frequency, mean, and SD were used. The Shapiro-Wilk test was also employed to examine the data distribution. Moreover, the Kruskal-Wallis test, the Chi-square test, and the Fisher’s exact test were utilized to compare the demographic variables between the four groups. Furthermore, the paired-samples t-test or the repeated measures analysis of variance (ANOVA) was employed to compare the mean scores in the dependent groups. The independent-samples t-test and the one-way ANOVA were also used for between-group comparisons. The SPSS Statistics software (version 19) was employed to conduct statistical tests, and the significance level was considered less than 0.05 with all the cases.

## Results

This study was completed using the Solomon four-group design with two control groups, as well as two intervention groups, one with and one without a pretest, the control group one and the intervention group one (with a pretest) and the control group two and the intervention group two (without a pretest).

In this study, a total number of 92 nursing and midwifery students (i.e., 64 nursing students and 28 midwifery students) participated in four groups of 23, in the form of two intervention groups and two control groups. Out of the participants in the four intervention and control groups, 10 were excluded from the study due to non-participation (Fig. 1).

The participants included 51 female students (62.2%) and 31 male counterparts (37.8%) with a total mean age of 21.23 ± 1.65 years. The mean and the SD of the age of the intervention groups one and two were 20.73 ± 0.86 and 21.28 ± 1.30 years, respectively. Additionally, the mean and the SD of the age of the control groups one and two were 21.75 ± 2.75 and 21.22 ± 0.94 years, respectively. Moreover, the Kruskal-Wallis test results did not show a statistically significant difference between the four groups in terms of age (x^2^ = 3.91; *p* = 0.271). The four groups did not differ with regard to other demographic variables (Table 2).

**Table 2:** Comparison of demographic variables between the four intervention and control groups

Variable	Variable levels	Intervention 1(n:23) N (%) or Mean (SD)*	Intervention 2(n:23) N (%)or Mean (SD)**	Control 1(n:23) N (%)or Mean (SD)*	Control 2(n:23) N (%)or Mean (SD)*	X^**2**^ or Fisher*	***P*** value
Age		20.73 (0.86)^**^	21.28 (1.30)^**^	21.75 (2.75) ^**^	21.22 (0.94) ^**^	3.91	0.271
Sex	Girl	12 (23.5)	12 (23.5)	15 (29.4)	12 (23.5)	2.758	0.430
	Boy	11 (35.5)	9 (29.0)	5 (16.1)	6 (19.4)		
Marital status	Single	21 (27.6)	20 (26.3)	18 (23.7)	17 (22.4)	0.817*	0.146
	Married	2 (33.3)	1 (16.7)	2 (33.3)	2 (16.7)		
living area	City	17 (23.9)	20 (28.2)	17 (23.9)	17 (23.9)	4.834*	0.183
	Village	6 (54.5)	1 (9.1)	3 (27.3)	1 (9.1)		
Indigenous	Yes	14 (28.0)	13 (26.0)	13 (26.0)	10 (20.0)	0.366	0.947
	No	9 (28.1)	8 (25.0)	7 (21.9)	8 (25.0)		
Residence while studying	Private house	3 (23.1)	4 (30.8)	4 (30.8)	2 (15.4)	0.948*	0.855
	Dormitory	20 (29.0)	17 (24.6)	16 (23.2)	16 (23.2)		
Field of Study	Nursing	16 (26.2)	16 (26.2)	16 (26.2)	13 (21.3)	0.692	0.895
	Midwifery	5 (33.3)	4 (23.8)	5 (19.0)	7 (23.8)		

The test used is the chi-square or Fisher’s exact test

for age Kruskal-wallis test was done

*value is fisher exact

**the value are Mean and standard deviation

The distribution of the pretest scores in both the intervention and control groups one, as well as in the posttest and the follow-up in all four groups was found normal. The PS *knowledge* scores before the intervention in the two groups of intervention one and control one were 15.60 ± 1.924and 16.65 ± 2.10, respectively, which were not significantly different (*p* = 0.098).

The results of the within-group comparisons revealed that the mean score of the PSKRE in the intervention and control groups one was different at three times. The post-hoc test results also established that the mean scores of the posttest and the follow-up were significantly higher than those of the pretest in the intervention group one (*p*-values in both cases were less than 0.001) and the mean score of the follow-up compared with that of the posttest showed a statistically significant descending trend (*p* = 0.006). In Control Group One, the post-test mean score (*p* < 0.001) was significantly higher than that of the pre-test, but there was no statistically significant difference between the mean scores of the pretest and the follow-up (*p* = 0.100). The follow-up mean score was significantly lower than that of the posttest (*p* = 0.003). In Intervention Group Two, the PSKRE mean score in the posttest and the follow-up was not statistically significant. However, the PSKRE mean scores for the Control Group Two, the intervention group, and the total control groups in the follow-up was significantly lower than that of the posttest (Table 3).

**Table 3:** Within group comparison of the average score of patient safety management before, immediately after and also 2 months

Group	Pre-test	Post-test	Follow up	t or F*	***P*** value
	Mean ± SD	Mean ± SD	Mean ± SD		
Intervention 1	15.60 ± 1.924	25.17 ± 3.32	21.60 ± 3.29	64.889*	< 0.001
Intervention 2	–	24.40 ± 3.67	23.38 ± 3.89	1.059	0.303
Control 1	16.65 ± 2.10	20.80 ± 4.16	16.10 ± 3.55	11.120*	< 0.001
Control 2	–	21.27 ± 3.86	17.05 ± 3.33	4.880	< 0.001
Total intervention	–	24.81 ± 3.47	22.33 ± 3.60	3.164	0.003
Total control	–	21.03 ± 3.98	16.55 ± 3.44	6.001	< 0.001

*Report statistics is *F* value

The PSKRE mean score in the control group two, the intervention group, and the total control groups in the follow-up was significantly lower than that in the posttest

The results of the between-group comparisons confirmed that the changes in the mean scores from the pretest to the posttest were different between the intervention and control groups one. The intervention group also showed a higher score increase. Furthermore, the changes in the PSKRE mean scores between the pretest and the follow-up were different in both intervention groups one and Control Group One, i.e., rising and falling trends in the mean scores in the intervention group and the control group, respectively (Table 4).

**Table 4:** Comparison of mean changes in patient safety management score before and immediately after the intervention and also before and 2 months after the intervention between control groups 1 and intervention 1

Time	Group (N)	Mean difference	Standard deviation	Mean difference				t statistic	Degree of freedom	*P* value
				Average	Standard error	Assurance interval for mean difference				
						Low limit	High limit			
**Post-test minus pre-test**	Intervention 1 (23)	9.57	2.83	5.41	1.28	2.77	8.05	4.204	41	< 0.001
	Control 1 (20)	4.15	5.12							
**Follow up minus before intervention**	Intervention 1 (23)	6.00	4.20	6.55	1.25	4.01	9.09	5.211	41	< 0.001
	Control 1 (20)	−0.55	4.01							

Test performed: Independent t

the PSKRE mean score in the control group two, the intervention group, and the total control groups in the follow-up was significantly lower than that in the posttest

The changes in the PSKRE mean scores from the posttest to the follow-up were not statistically significant between the four study groups (Table 5). In addition, the changes in the mean and the SD from the post-test to the follow-up in the two groups of intervention and total control were 2.48 ± 5.15 and 4.47 ± 4.49, respectively. The two groups did not have a statistically significant difference in terms of changes in their mean scores (t = 1.89; *p* = 0.071).

**Table 5:** Comparison of mean changes in safety management score immediately after and 2 months after intervention between the four groups

Time	Group (N)	Average changes	Standard deviation	F	***P*** value
Follow minus post-test	Intervention 1 (23)	−3.57	4.91	1.941	0.130
	Intervention 2 (20)	−1.25	5.28		
	Control 1 (20)	−4.70	5.38		
	Control 2 (18)	−4.22	3.67		

Test performed: one-way analysis of variance

The results of the between-group comparisons confirmed that the changes in the mean scores from the pretest to the posttest were different between the intervention and control groups one

## Discussion

The main purpose of this study was to compare the effects of PS education using the FC through NPE and the conventional methods on knowledge retention associated with PS in nursing and midwifery students. The study results showed that the integration of the FC and the NPE compared with the conventional methods had broadened the levels of knowledge in the students in the field of PS. However, PS education through the FC through NPE was not significantly different from the conventional methods, in terms of their effects on knowledge retention among nursing and midwifery students, 2 months following the intervention.

The results of the within-group comparisons showed that both FC through NPE and conventional methods could boost learners’ knowledge. The between-group comparison results also demonstrated a greater increase in learning in the group receiving the FC through NPE and the four-group Solomon design confirmed these findings. It was established that more learning took place in the intervention group than in the control group, even in terms of the effects of the pretest.

Limited research has so far examined the integration of the FC with other methods [3, 25, 26] and there was no research available, to the best of the authors’ knowledge, reflecting on the FC through NPE. Therefore, the study findings were compared with some investigations that had explored the NPE, as well as the FC, independently. The study findings were consistent with those by, Shohani et al. (2020), Kim et al. (2019), Guraya et al. (2020), on NPE) [12, 27, 28], and Hu et al. (2018), Li et al. (2020), Charles-Ogan G, Williams (2015) and Chu et al. (2019) on using the FC [18, 29–31]. However, these findings were not in line with the study by Hatami-Rad et al. (2017) and Sevenhuysen et al. (2014) on NPE [32, 33]. Perhaps the reason for the inconsistency of the results was that, an integration of the FC and NPE was used in the present study, leading to greater effectiveness of education and a difference between the intervention and the control groups in terms of the PSKE mean scores. It is believed that using a teaching method based on an educational theory alone cannot promote learning, so an integration of pedagogical methods should be used to create active learning thinking in learners [34]. Another point was that, the levels of knowledge in students were evaluated in the present study, while in the mentioned investigations, clinical skills and performance had been appraised. The complexity and the specific difficulties of clinical skills [27] could be thus a reason for the inconsistency of the findings. Further studies could also determine the effects of similar pedagogical interventions on different levels of knowledge.

On the other hand, the present study was not in agreement with the reports by Whillier et al. (2015) and Harrington et al. (2015) on using the FC [5, 27]. In this sense, Whillier et al. (2015) found that levels of satisfaction in students, as well as their mean scores in terms of knowledge in the FC and structured classrooms instructed by teachers through problem-solving and case study were similar. The reason for the difference could be related to the educational content. In the present study, the PS program was presented through the FC as governed by NPE, since it was one of the most important dimensions of quality of care and any harm to patients was in conflict with the philosophy of health care. In the PS education, the description of natural events and interesting, objective, and tangible scenarios could cause the content not to be presented in a soulless way. Therefore, learners’ attention could be more drawn to the educational content, and it could become more comprehensible to them.

Whillier et al., on the other hand, had used the FC to teach neuroanatomy [35]. Although this approach was somewhat self-directed learning [36, 37], it might not be very suitable for biomedical courses and those with heavy content. Of course, the effect of new pedagogical methods on different contents needs further investigation. The difference between the two studies might be also related to the conventional methods with which the new method was compared. In the study by Whillier et al. (2015), the content was provided for the students on a web-based source before teaching began via the conventional methods. This is while in the present study, the content was provided for the students at the same time as the workshop program started using the conventional methods. It is important to note that most of the lessons were taught in a traditional education context, where this study was conducted (Iran) and there was little use of new methods such as the Internet to upload content and the NPE or the FC. This novelty could thus increase the attractiveness of the method and make it more effective. In general, based on the study results, the integration of the two new methods of teaching could make the education of PS topics more effective than the conventional methods. The FC is also technology-dependent, and students plan for their learning based on their different learning abilities and styles [38, 39]. This feature, along with the more attractiveness of the method, the bolder role of students in learning, as well as the less stress because of the NPE can be among the reasons for the greater impact of the integrated method of the FC and the NPE. However, these results cannot be generalized to teaching other courses, because it does not mean that integrating the FC and the NPE is necessarily the most effective strategy. Accordingly, it is suggested that enough space and facilities be provided for the use of web-based education, as well as the infrastructure for the practice of modern teaching methods along with the integration of these methods at universities. In addition, the required conditions should be put on the agenda of universities for examining the effect of new methods to determine the most appropriate ones for course contents.

The study findings showed that this pedagogical method had no effect on students’ knowledge retention after 2 months of education compared with the conventional methods. However, the PSKRE mean scores 2 months after the intervention were still higher in the two intervention groups than the control group, but the drop in the mean scores from the posttest to the follow-up was similar in the four study groups. Javaheri et al. (2018) in their study of the effect of peer education and Graham et al. (2019) and Gu et al. (2020) by examining the impact of the FC further confirmed that the levels of knowledge at the follow-up were higher in the intervention group, a finding which is in line with the present study [3, 38, 39]. These studies had not measured the changes in the mean scores from the posttest to the follow-up, which could indicate a decrease in knowledge over time as a better criterion for assessing retention. The present study revealed that knowledge loss after the posttest was similar in the intervention and control groups. In fact, the higher mean score of the PSKRE follow-up was the output of more learning and a higher knowledge score immediately after the intervention. Therefore, examining the decline in learning over time could lead to a better and more explicit understanding of the impact of new methods of teaching and the integrated ones and this help teachers decide to select the most appropriate methods. Deep learning can further stabilize the acquired knowledge and lead to academic achievement [40]. Therefore, it is important to study knowledge retention across different pedagogical methods. The point is that learning and knowledge retention are part of the issues that should be measured in modern teaching methods and then compared with conventional ones. In addition, students’ satisfaction with teaching methods and their attractiveness are among the issues that should be considered when using different pedagogical methods. *In the end, it has to be noted that the mean loss of knowledge from the posttest to the follow-up was numerically lower* in the intervention group*;* however, the interval range was wide *which can be due to the small sample size* of this study*. Repeating a study with a larger sample size could produce* more accurate results.

Unfortunately, the present study suffered from some limitations. For example, it did not assess student satisfaction, so it is suggested that it be considered in future studies. Another limitation of this study was the comparison of a new method integrated with the conventional one, which did not determine whether the integrated method was more effective than the two new methods of the FC and the NPE alone. As with most pedagogical interventions, it is possible to exchange information between the control and intervention groups, which constitutes another limitation of this study. Motivation, experience, and attitude are relevant in the comparison of groups, but the present study did not consider these confounding *variables*. Therefore, the achieved result cannot be associated with only the teaching method. Thus, *it is recommended that these variables be taken into account in future studies.*

## Conclusion

The findings of the present study showed that the use of the FC through NPE could increase the PSKRE mean scores and thus boost learning, but, in general, it had no effect on knowledge retention associated with PS in nursing and midwifery students. Given the limited number of studies in this field, it is recommended that further research be conducted with larger sample sizes in other fields and disciplines of medical sciences, and additionally examine students’ levels of satisfaction with different teaching methods.

## Data Availability

The anonymized datasets used and/or analyzed during the current study are available from the corresponding author on reasonable request.

## Abbreviations

FC: Flipped Classroom
FC through NPE: Flipped Classroom through Near Peer Education
NPE: Near Peer Education
PS: Patient safety
PSKRE: Patient Safety Knowledge Retention Exam
RCT: Randomized controlled trial
WHO: World Health Organization
